# Chromosomal integration of HHV-6A during non-productive viral infection

**DOI:** 10.1038/s41598-017-00658-y

**Published:** 2017-03-30

**Authors:** Nitish Gulve, Celina Frank, Maximilian Klepsch, Bhupesh K. Prusty

**Affiliations:** Biocenter, Chair of Microbiology, University of Würzburg, 97074 Würzburg, Germany

## Abstract

Human herpesvirus 6A (HHV-6A) and 6B (HHV-6B) are two different species of betaherpesviruses that integrate into sub-telomeric ends of human chromosomes, for which different prevalence rates of integration have been reported. It has been demonstrated that integrated viral genome is stable and is fully retained. However, study of chromosomally integrated viral genome in individuals carrying inherited HHV-6 (iciHHV-6) showed unexpected number of viral DR copies. Hence, we created an *in vitro* infection model and studied retention of full or partial viral genome over a period of time. We observed an exceptional event where cells retained viral direct repeats (DRs) alone in the absence of the full viral genome. Finally, we found evidence for non-telomeric integration of HHV-6A DR in both cultured cells and in an iciHHV-6 individual. Our results shed light on several novel features of HHV-6A chromosomal integration and provide valuable information for future screening techniques.

## Introduction

Human herpesvirus 6 (HHV-6A and HHV-6B) belongs to the betaherpesvirus family and is a unique human pathogen as it is the only known pathogen that integrates into telomeric ends of human chromosomes^1–3^. Chromosomally integrated HHV-6 (ciHHV-6) has been detected only in human sub-telomeric ends suggesting viral integration through homologous recombination between telomeric repeat sequences present at the end of human chromosomes and HHV-6 genome. When integrated into germ cells, HHV-6 can be inherited (iciHHV-6) thereby ending up in 1 or more copies of viral genome in every nucleated cell of the body^4, 5^. Even after several years of research on viral integration, our knowledge is still limited. There are several open questions including (a) is viral integration dependent upon host cell physiology rather than a viral protein? (b) are integrated viral genomes always stable ? and (c) does the iciHHV-6 genome maintain its integrity in all cells ? In this paper, we followed an *in vitro* approach to answer some of these questions. Our results show evidence for both genome integration as well as unusually high stability of HHV-6A direct repeat (DR) elements. We also show evidence for non-telomeric integrations of HHV-6A in various cells where viral integration is possibly mediated by non-telomeric repeat sequences.

## Results

### Additional viral DR are often detected in iciHHV-6 individuals

**Figure 1:**
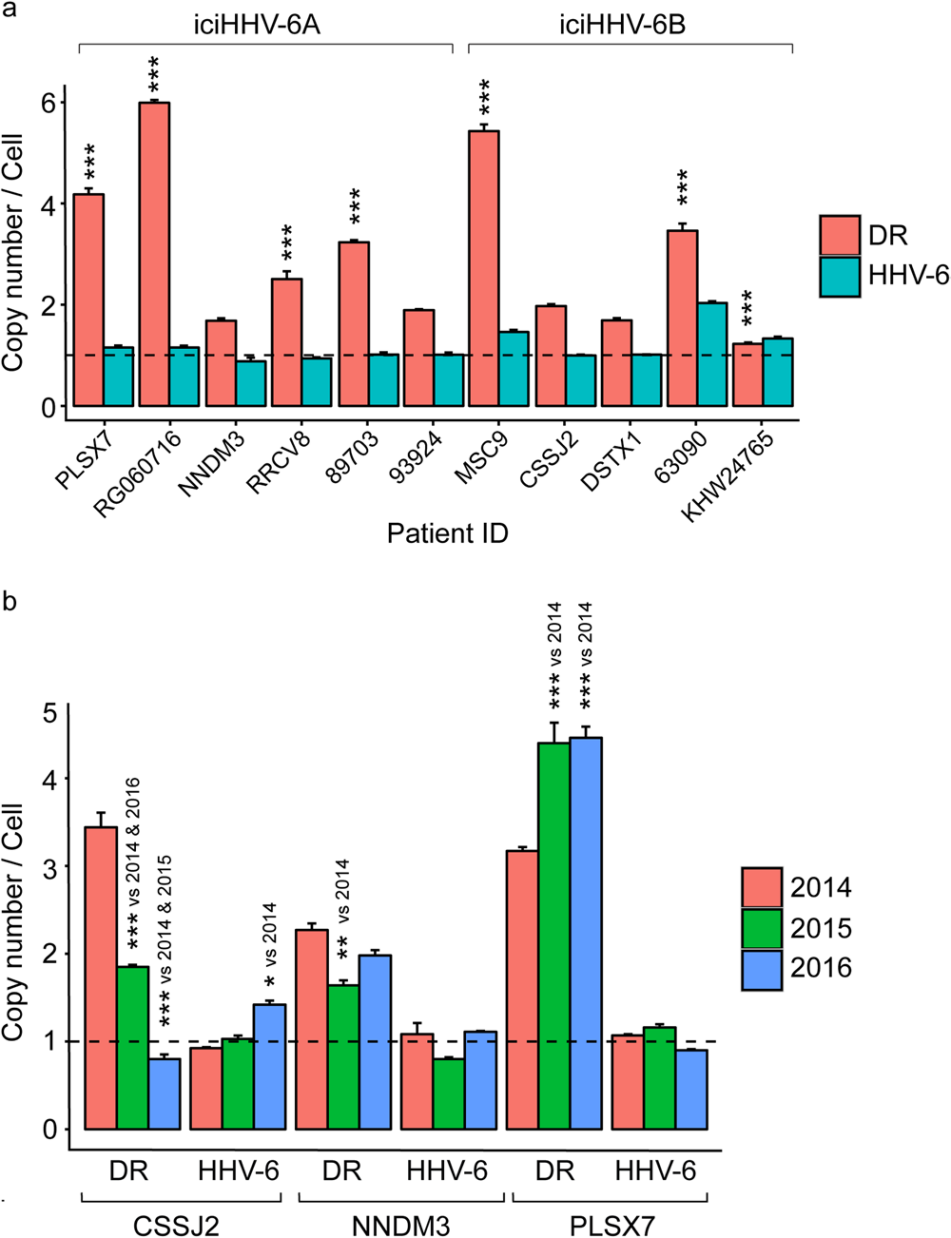
High number viral DRs are detected in many iciHHV-6 individuals. (**a**) Number of copies of HHV-6 genome and viral DR per cell were quantified using qPCR. HHV-6 and DR copy numbers for different individuals were compared against patient 93924 for statistical analysis as 93924 had ~1 copy of HHV-6A and ~2 copies of DR representing the most ideal scenario. (**b**) Viral genome copy numbers and DR copy numbers were quantified over a period of 3 years in 3 different iciHHV-6 individuals. Data represents mean values of 3 different biological triplicates. Statistical analysis was done by using two-way Analysis of Variance (ANOVA) followed by Tukey’s Honest Significance Differences (HSD) test for determination of individual comparisons, adjusted P values. * < 0.05, ** < 0.01, *** < 0.001. Copy number of 1 is marked as a baseline (dotted line).

HHV-6 DRs are ~8 kb long and contain regions of highly repetitive DNA including telomeric repeat sequences. We have previously shown occurrence of extra-chromosomal viral DRs in iciHHV-6 individuals^6^ indicating the possibility that one of the viral DRs is lost after viral integration. Similarly viral excision from the human chromosome might lead to retention of a part of viral DR in the human genome in the absence of rest of the viral genome. As DRs carry telomeric repeat sequences and are crucial for telomeric integration and activation of HHV-6, we analysed the ratio of viral DR to viral genome in total DNA derived from peripheral blood mononuclear cells (PBMCs) from 11 iciHHV-6 individuals (6 iciHHV-6A and 5 iciHHV-6B). iciHHV-6 status of these individuals were confirmed by positive detection of viral genome in hair follicles and by detection of integrated viral genome in PBMCs by fluorescence *in situ* hybridization (FISH). Freshly isolated PBMCs derived from peripheral blood were used for all the studies. Highly specific PCR primer sets were developed to differentiate HHV-6A DR from HHV-6B DR and their quantitative measurement (see supplementary Table S1 and Fig. S1). Against the expected 1:2 ratio of HHV-6 genome to viral DR, we observed increased number of viral DRs in several iciHHV-6 individuals (Fig. 1a). We detected up to 5–6 copies of viral DR against one copy of the viral genome in 3 of the samples. Furthermore we analysed DR-to-viral genome ratio in total DNA isolated from PBMCs derived from freshly drawn blood from 3 of these individuals over a period of 3 years (once per year) and found almost constant viral copy numbers in all the 3 samples (Fig. 1b). In one of the individuals, the number of viral DR copies remained almost constant whereas it varied significantly in the other two iciHHV-6 individuals over a period of 3 years. These studies suggest that the viral DR may behave differently than rest of the viral genome within germline chromosomally integrated iciHHV-6 individuals.

### HHV-6A genome is lost during non-productive viral infection

**Figure 2:**
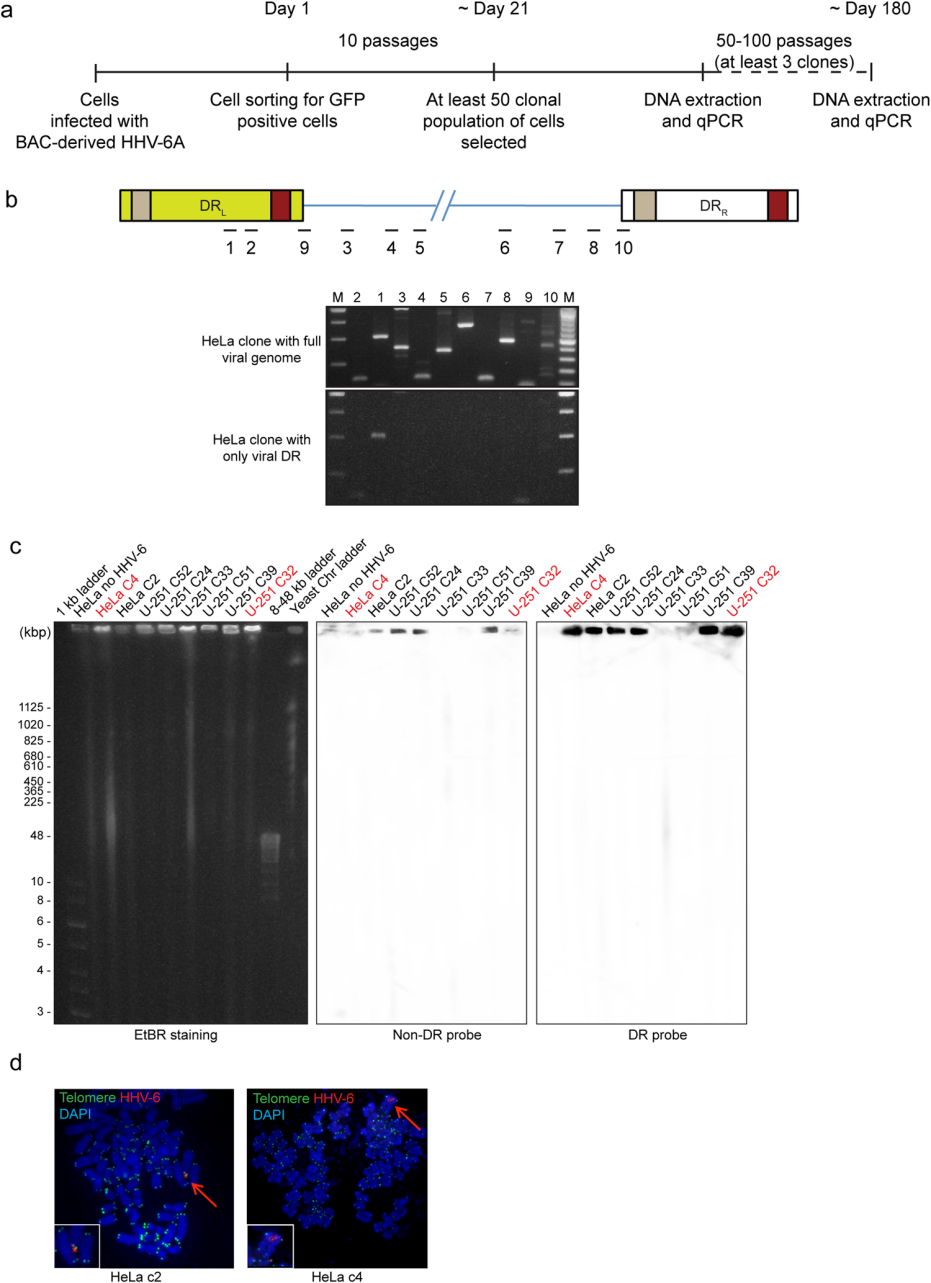
*In vitro* cell culture infection model to study chromosomal integration of HHV-6. (**a**) Diagrammatic representation of the experimental set up. (**b**) 10 different regions of HHV-6A genome in 2 different clones of HeLa cells were tested by conventional PCR. Upper panel represent approximate location of primer pairs within HHV-6A genome. Lower panel shows ethidium bromide stained agarose gel images showing amplified PCR products. M, 1 kb DNA ladder; 1, DR6; 2, DR7; 3, U22; 4, P41; 5, U42; 6, U79; 7, U83; 8, U91; 9, DRL junction region; 10, DRR junction region. (**c**) Presence of chromosome associated full-length viral genome and viral DR was studied in various HeLa and U-251 clones by PFGE. Blots were first hybridised with a probe that does not detect HHV-6 DR (non-DR probe), stripped and then re-probed with a DR specific probe (DR probe). Cells positive for viral DR alone are indicated in red. (**d**) FISH analysis of integrated HHV-6A in HeLa cells. Left panel shows integrated viral genome in clone 2. Co-staining with telomere specific probe is indicated. The right panel shows the integration of viral DR in clone 4. HHV-6A DR specific probe is used for viral genome detection. Arrowhead indicates integrated viral genome, which is also shown as enlarged images on lower left hand corner of each image.

In order to understand the process of viral integration and fate of integrated viral genome, we followed an independent, but similar *in vitro* approach as shown in some recent publications^3, 7^. However, instead of looking into integration of the viral genome, we analysed retention of viral genome (integrated as well as non-integrated) after viral infection. As it was crucial to start with a pool of 100% infected cells that will assure us of the fact that loss of the viral genome or the GFP gene from the BAC during subsequent passaging is not because of lack of infection, we utilised BAC-derived HHV-6A^8^ that expresses GFP protein in infected cells. Initial trial experiments in HeLa cells showed complete loss of GFP protein expression within 4–5 days of first infection indicating possible chromosomal integration or loss of viral genome from these cells. Hence, we sorted GFP positive HeLa cells within 24 h after the first infection and generated at least 50 clonal populations of cells. Presence of the viral genome was quantified in these cells after ~10 passages by qPCR using a primer pair designed against HHV-6 U94 ORF (Fig. 2a). In parallel, viral DR copy numbers were also quantified in these cells. Similar experiments were also carried out in 2 different telomerase positive tumor cells (ovarian (SK-OV-3)^9^ and glioblastoma (U-251))^10^ to compare integration of HHV-6A in different cells. 35–50 clonal populations were analysed from all the three cells. In HeLa cells, 51.4% of the clonal populations retained variable number of viral genomes per cell ranging between 1–7 copies whereas DR copy numbers were in the range of 2–27 copies (Table 1). Interestingly, 5 (14%) clones retained mostly viral DR in the absence of the rest of the viral genome (Supplementary Fig. S2a, Table 1). 34% of the HeLa cells lost the viral genome completely within first 10 passages. In SK-OV-3 cells, 80% of the cells lost the viral genome completely (Supplementary Fig. S2b). In rest of the 20% of the clones, viral genome was gradually lost showing less than 0.5 copies of viral genome per cell. In 12% of the cells, viral DR was retained whereas most of the viral genome was gradually lost. Only 4 (8%) of the cells retained more than 1 copy of the viral genome. Interestingly, in U-251 cells 17 (32%) of the clones retained more than 1 copy of DR alone in the absence of rest of the viral genome (Supplementary Fig. S2c). Two of the clones retained full-length viral genome. It is noteworthy that our experimental set up was designed to analyse cells after 10 passages of first infection in order to understand viral integration during early infection. Hence, it was evident that we detected viral copy numbers in clonal population of cells that had less than 1 copy of ciHHV-6A per cell showing loss of viral DNA during subsequent growth phase of the cells.

**Table 1:** Differences in loss of viral genome in different cell lines as studied by qPCR analysis of HHV-6A genome equivalents and DR equivalents in different clonal population of cells.

Cells	Total number of clones analysed	Number of clones that retained HHV-6A genome together with DR	Number of clones that retained DR alone	Number of clones that lost most of the HHV-6A genome including DR
HeLa	35	18 (51.4%)	5 (14.2%)	12 (34.28%)
SK-OV-3	50	4 (8%)	6 (12%)	40 (80%)
U-251	52	2 (3.84%)	17 (32.69%)	33 (63.46%)

Presence of viral DR alone was verified by conventional PCR amplification of at least 10 different regions of the viral genome in both HeLa clone 2 and clone 4 (Fig. 2b). Presence of viral DR alone in the absence of amplification of DNA from various viral ORFs was confirmed in clone 4. Primers designed at the junctions of DR and viral ORFs also failed to amplify in these clones suggesting retention of DR alone in these cells.

Quantification of viral genome equivalent per cell is not informative about the status of viral integration. Moreover, it is important to study if the DR alone is retained in an integrated state or as an episome in cells after viral infection. Hence, we followed 2 independent approaches. We carried out pulsed-field gel electrophoresis (PFGE) exclusively to see if the viral genome remains chromosome-associated or not. In parallel, we designed a modified approach of inverse PCR (iPCR) to amplify the junction region of DR-T2 at the end of the virus genome with the human chromosome (integration) or with other virus sequences (concatamer or episome), combined with Southern hybridisation using four different probes, to identify various different forms of viral genome (telomeric integrated^11^, concatemeric viral DNA^12–15^, small circular extra-chromosomal DR DNA^6^ as well as circular viral DNA having single DR^6, 16^). Results obtained from both these approaches, when combined together, provided a clear idea about location and composition of viral genome after *in vitro* non-productive viral infection.

**Figure 3:**
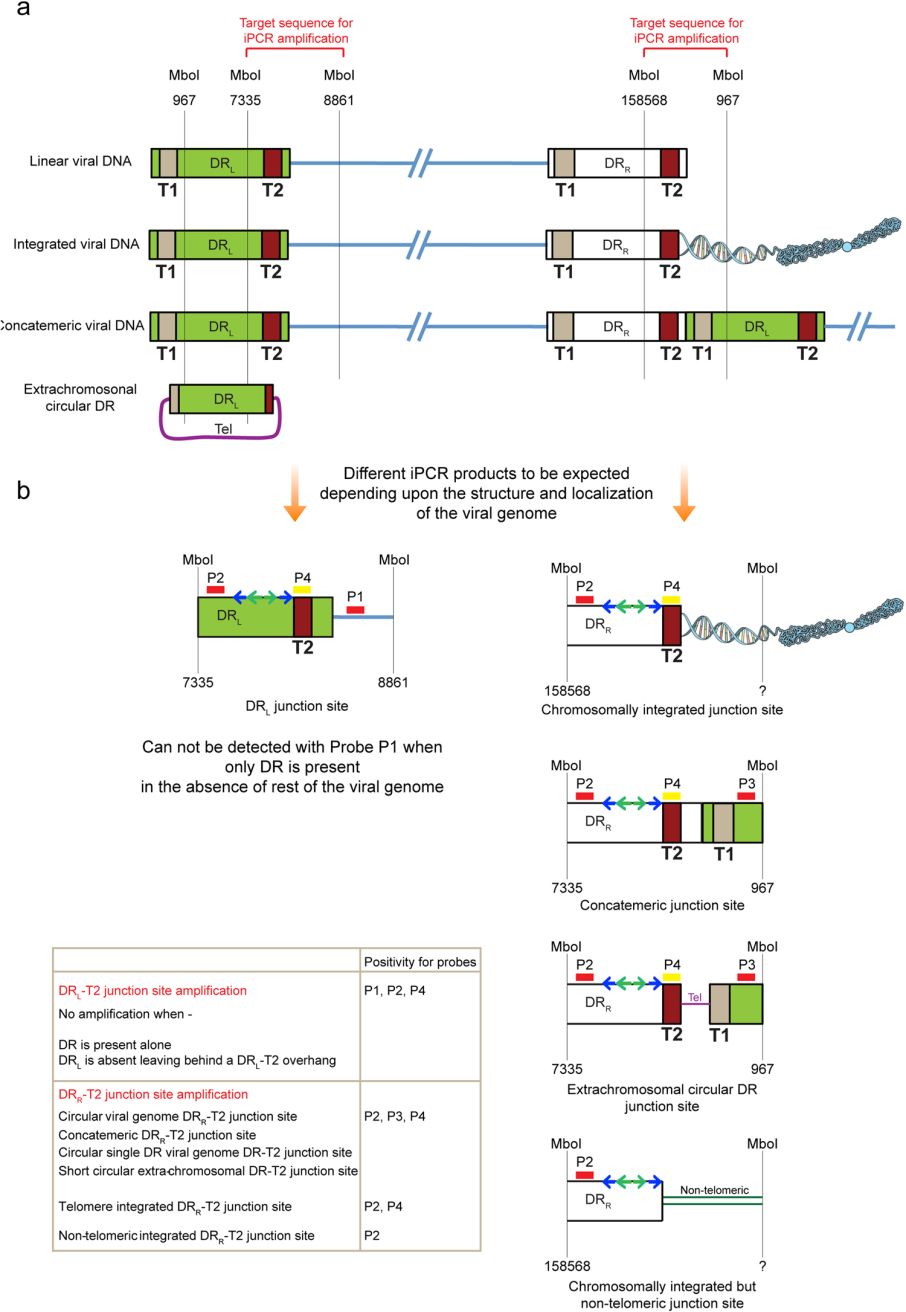
Schematic representation of the inverse PCR experimental set up to amplify DR-T2 junction region. (**a**) Various conformations of HHV-6 genome reported so far are compared on the backdrop of the inverse PCR. MboI is a frequent cutter restriction enzyme. Hence, only the cut sites of our interest are indicated. Nucleotide position of the MboI cut sites (reference genome sequence X83413.1) is indicated. DR_L_, left direct repeat; DR_R_, right direct repeat; T1, heterogeneous telomeric repeat sequence; T2, homogeneous telomeric repeat sequence. (**b**) Different combinations of iPCR products expected are described depending upon the structure and integration status of the viral genome. Locations of the 4 hybridisation probes (P1-P4) are marked. First sets of inverse primers are indicated with blue arrows and nested primer pairs are indicated with green arrows. A combination of probes that can detect specific viral structures is indicated within a Table.

We found chromosome associated viral DNA in all the three cells that were positive in qPCR (Fig. 2c, Supplementary Fig. S3a), whereas some of the clonal populations of SK-OV-3 cells carried both chromosome-associated as well as extra-chromosomal viral DNA (Supplementary Fig. S3b). We also confirmed qPCR results where only chromosome associated viral DR was detected in some of the cells (HeLa c4 and U-251 c32 in Fig. 3c). We detected low copies of extra-chromosomal viral DR in some of the HeLa clones (Fig. S3a). Presence of chromosome associated full-length viral genome as well as DR alone was also verified by FISH analysis in HeLa clone 2 and clone 4 respectively (Fig. 2d) using viral genome or DR specific FISH probes. We could not detect multiple integration sites of DR by FISH in several cells that carried more than two DR copies. This might be because of use of shorter DR FISH probes (~3, 5 kb), which is difficult to detect using a fluorescence microscope. It is also possible that the viral DRs are mostly in concatemeric form in cells that carry higher copies of DR, which cannot be seen as separate signals using FISH. However, we managed to see a few clonal populations of cells like U-251 clone 15 showing more than one DR signal in FISH Fig. S3c. In addition, we also detected more than one DR integration site in one of the iciHHV-6A patient (PLSX7) (Supplementary Fig. S3c).

In order to understand the stability of the integrated viral genome during continuous passaging, we cultured a few clones from each cell for at least 9–10 passages and DNA samples were collected in between to check the viral copy numbers. Results were variable for different sets of cells. Interestingly, in one of the U-251 clones tested, the viral copy number remained the same with minor variation during the later passaging of cells whereas viral DR numbers constantly changed during continuous growth phase (Supplementary Fig. S4a). Under similar culture conditions, one of the DRs was lost during early passaging and rest of the genome with the other DR remained stable in a SK-OV-3 clone (Supplementary Fig. S4b). In a second set of SK-OV-3 cells, we did not see any change in viral or DR copy numbers (Supplementary Fig. S4c).

**Figure 4:**
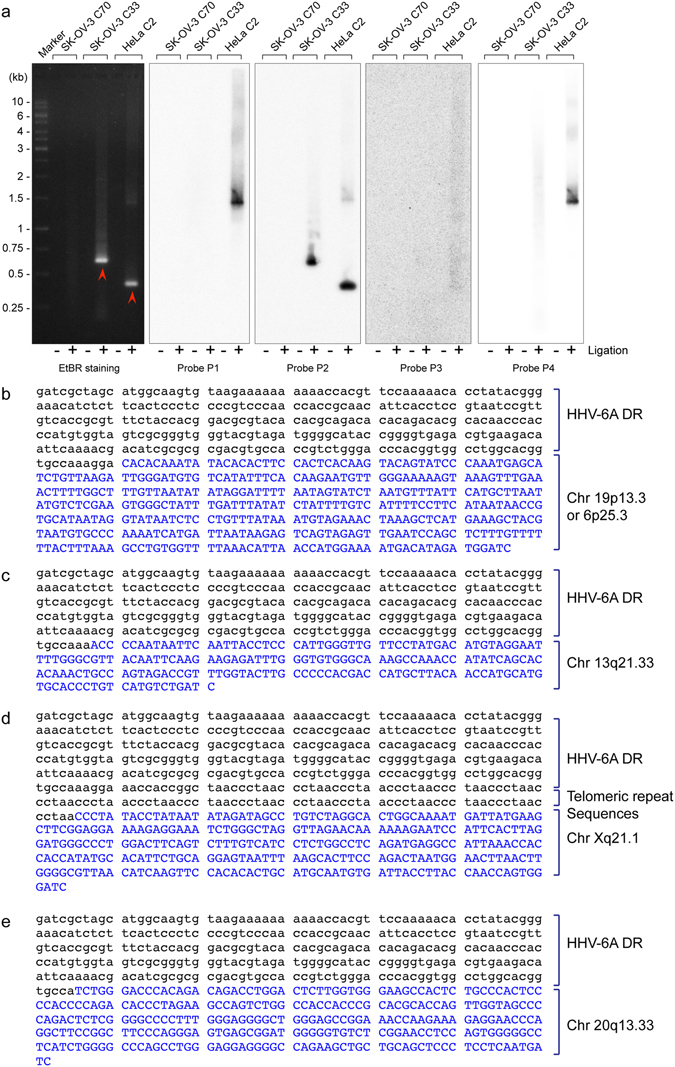
Non-telomeric chromosomal integration of HHV-6A. (**a**) iPCR products from three different cells were checked by Southern hybridisation using 4 different probes as explained in Fig. 3. Potential bands that did not hybridise with a telomere probe (Probe P4) are marked with red arrowhead and were processed for sequence identification. (**b**–**e**) Sequencing and identification of non-telomeric junction site of HHV-6A in four different clonal populations of cells. (**b**) Sequence analysis of junction site of HHV-6A in SK-OV-3 clone 33. (**c**) Sequence analysis of junction site of HHV-6A in HeLa clone 2. (**d**) Sequence analysis of junction site of HHV-6A in HeLa clone 4. (**e**) Sequence analysis of junction site of HHV-6A in U-251 clone 15. Viral DNA sequences are marked in black whereas chromosomal DNA sequences are marked in blue.

We devised a reproducible and elaborate approach of iPCR to understand the retention of full-length or partial viral genome. A schematic representation of the method and various different forms of viral genome that can be detected by this approach is described in Fig. 3. This approach specifically identifies chromosomal integration junction sites near DRR-T2 and differentiates it from the same originating from DRL-T2, concatemeric viral DNA as well as viral DNA that are circular and carry a single DR. We successfully utilised iPCR to verify several clonal populations of cells that predominantly showed presence of viral DR alone by qPCR (U-251 clone 15 and 39 in Fig. S5a, HeLa clone 4 in S5b). We detected a combination of result for SK-OV-3 clone 33 in iPCR that suggested chromosomal integration of HHV-6A at DRR-T2 where possibly DRL was lost, thereby leaving behind a single DR (Fig. 4a, supplementary Table S2). Such a combination of integrated viral genome has been described before^6^. We also detected concatemeric viral DNA in U-251 clone 15 and clone 39 corroborating our FISH results (Supplementary Fig. S3c). In summary, our *in vitro* assays detected an unusual situation of viral integration where DR alone can be retained in the absence of rest of the viral genome in addition to complete genome integration.

### HHV-6A integrates into non-telomeric chromosomal locations

In the previously described iPCR approach, we detected several bands originating from DR-T2 that did not hybridise with a telomeric probe (SK-OV-3 clone 33 in Fig. 4a, U-251 clone 39 and HeLa clone 4 in Supplementary Fig. S5a and b respectively). This may be because of presence of a very short telomeric repeat sequence at the junction site. However, it is also possible that these viral DRs are integrated into a non-telomeric chromosomal site. Sequencing of these PCR products revealed unique non-telomeric integration sites of ciHHV-6A at the chromosomal location 19p13.3 or 6p25.3 in SK-OV-3 clone 33 (Fig. 4b), at Chr 13q21.33 in HeLa clone 2 (Fig. 4c), at Chr Xq21.1 in HeLa clone 4 (Fig. 4d) and at Chr 20q13.3 in U-251 clone 15 (Fig. 4e). The identified sequence of chromosomal integration sites for SK-OV-3 clone 33 (Fig. 4b) has 100% homology to two different chromosomal ends (19p13.3 or 6p25.3) making it difficult to specify the location within repetitive sequences. Interestingly, ciHHV-6A DR integration was observed within the intronic region of human G-alpha interacting protein isoform B (GAIP) in U-251 clone 15 (Supplementary Fig. S6a). Moreover, 3 of the non-telomeric integration sites identified in our experimental set up did not contain typical telomeric repeat sequences.

**Figure 5:**
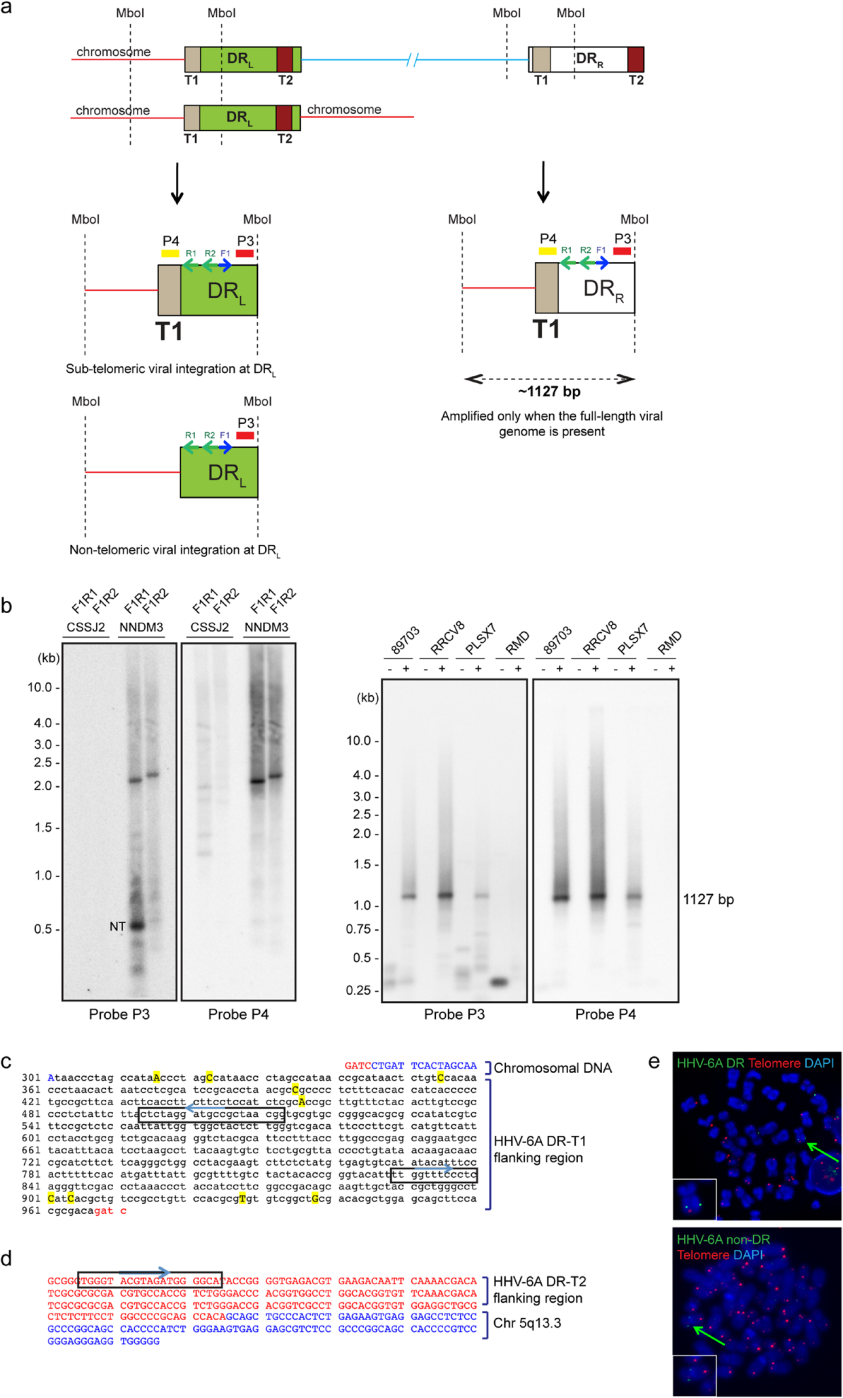
iPCR identified non-telomeric chromosomal integration of HHV-6A in iciHHV-6 individuals. (**a**) Schematic representation of iPCR to amplify DR-T1 junction site. Different combinations of iPCR products expected are described depending upon the structure and integration status of the viral genome. Locations of the 2 hybridisation probes (P3 and P4) are marked. F1, forward primer; R1 and R2, two reverse primers. (**b**) iPCR products from 4 different iciHHV-6A individuals (NNDM3, PLSX7, RRCV8 and 89703), a iciHHV-6B individual (CSSJ2) and non-HHV-6 control (RMD) were analysed by Southern hybridisation using two different probes. ~1.1 kb amplimer is expected from a full-length viral genome irrespective of integration status. CSSJ2 was used as a negative control to check primer specificity. NT, possible non-telomeric integration. (**c**) Sequence analysis of non-telomeric junction site at HHV-6A DR-T1 in sample NNDM3. (**d**) Sequence analysis of non-telomeric junction site at HHV-6A DR-T2 in sample NNDM3. Sequences within the rectangular boxes mark the primer locations. (**e**) FISH analysis using PBMCs from NNDM3 sample shows one of the non-telomeric integration sites. Both DR specific probe as well as a non-DR probe was used for HHV-6 detection. Arrowhead indicates integrated viral genome, which is also shown as a zoomed image on lower left hand corner of the image.

This prompted us to look for such non-telomeric integration sites of ciHHV-6 in iciHHV-6 individuals. We theorised that potential non-telomeric integration sites would have 2 chromosomal junction ends (right and left) unless the chromosome is broken at one end or has a telomeric end. For this reason, we changed our iPCR approach and designed a set of inverse primers that can amplify the left end of DR (Fig. 5a). Amplification of desired product at the left end of the DR is only possible if the viral genome is integrated using DR-T1 of the viral genome (Fig. 5a). As expected we did not see any amplicons arising from the left end of the genome in most of the iciHHV-6 individuals (right panel of Fig. 5b), supporting the notion that HHV-6 is mostly integrated using its right terminus (DRR-T2). However, we cannot avoid the facts that mismatched primers or possible large size of amplicons may not allow amplification of the DNA during the iPCR. However, in one of the iciHHV-6A individuals we detected a unique amplicon that did not hybridise with a telomeric probe (left panel of Fig. 5b). In addition, two different sets of reverse primers were used in iPCR from which only one resulted in amplification. Sequencing of this PCR product revealed another unique non-telomeric chromosomal integration site, which could not be mapped precisely due to its short sequence length and sequence similarity to multiple chromosomes (Fig. 5c). Hence, we used the previous set of iPCR primer designed near DR-T2 (Fig. 3) and identified the integration site as chromosome 5q13.3 (Fig. 5d). In addition, we observed extensive sequence variations at the junction region of viral genome (Fig. 5c) that might explain the lack of amplification with one of the reverse primer (R2). In depth sequence analysis of the integration site suggested viral integration into the intronic region of human angiogenic factor with G Patch and FHA Domains 1 (AGGF1) (Supplementary Fig. S6b), which plays a key role during angiogenesis by regulating proliferation of endothelial cells^17^. Non-telomeric integration of HHV-6A in this iciHHV-6 individual was further validated by FISH analysis using a DR- specific and a non-DR probe (Fig. 5e). These results corroborated our finding of non-telomeric integration of HHV-6A in our *in vitro* assays.

## Discussion

HHV-6A and HHV-6B genomes integrate into human sub-telomeric ends where integration is facilitated via typical telomeric repeat sequences^6, 11, 16^. Due to the absence of elaborate *in vitro* studies on the integrated viral genome, it is generally accepted that almost full-length viral genome is integrated into human chromosomes. Several *in vivo* sequencing studies in over 50 iciHHV-6A/B individuals have demonstrated genome integration, including 5 complete genomes and over a dozen subtelomere integration sites determined^5, 11, 16, 18, 19^. Most of the PCR-based epidemiological studies utilise primer pairs designed against viral ORFs to detect and quantify viral copy numbers. It is also accepted that integrated viral genomes possess either 1 or more copies of viral DR per genome^16, 18^. However, recent studies have detected abnormalities in viral genome composition^4, 20^ showing either decreased or increased number of viral DRs in iciHHV-6 individuals. We studied viral DR copy numbers using gene-specific primers against both HHV-6A and HHV-6B and found similar alterations in viral DR copy numbers in both iciHHV-6A and iciHHV-6B. Our *in vitro* infection data shows possibilities of loss of viral genome leaving behind the viral DR(s) integrated in the genome. We have previously shown that host cell telomeric circle (t-circle) as one of the possible means of viral excision^6^ and hence viral activation can be dependent upon the available mode of telomere maintenance within the cell. The unique observation of sole DR retention in all the 3 cells studied supports our previous notion that immediately after infection and integration, viral genome undergoes excision possibly through t-circle formation^6^ thereby leaving behind a part of the viral DR integrated in the genome. Even though cells in continuous culture might have multiple methods of telomere maintenance (telomerase-dependent as well as -independent) and can behave differently than *in vivo* germ cells, similar situations of viral genome loss and retention of DR may arise during germ line infections. Our results hence argue for detection of viral DR together with any other viral ORF as an important step for the study of HHV-6 integration.

In many of the iciHHV-6 donor PBMCs as well as cultured cells we quantified more than 2 copies of viral DR against one copy of the genome. However, this does not always imply that the viral genome reintegrates several times to have multiple copies of DR. We, together with others, have shown previously^6, 16^ that DR can be detected as a short circular DNA molecule in HHV-6 infected cells. Such circular viral DRs have the possibility to replicate themselves using telomeric DNA as a template^21^ and can also possibly reintegrate as a concatemeric DNA. This process can lead to an increase in viral DR numbers without having increased number of multiple DR integration sites. Our results also show that loss of viral genome is a frequent process in all the cells studied so far. Recently, loss of iciHHV-6 genome has been reported in a primary effusion-like lymphoma sample^19^ suggesting a role of viral activation in tumour development or a mere co-incident. Our *in vitro* infection data supports similar *in vivo* conditions where the viral genome can be lost during continuous cell growth or reactivation. It would be interesting to study the retention of viral DR in these *in vivo* cases and their potential role in tumor development. Viral DR is a unique region of the viral genome that encodes proteins like DR6 that interacts with p53^22, 23^ and hence has been predicted to have tumorigenic potential^24^. Several viral miRNAs are also expressed from HHV-6 DR^25^ thus supporting the role of viral DR in potential disease progression. Hence it is plausible that DR alone could also contribute to changes in host cell physiology. Our study is primarily focused on ciHHV-6A. However, it has been shown that chromosomal integration frequency might vary between iciHHV-6A and iciHHV-6B^5^. Hence it will be interesting to see if ciHHV-6B can also show similar characteristic features like ciHHV-6A.

Our results, as a pioneering study, show possibilities for non-telomeric integration of ciHHV-6A. We have identified at least one such integration site in all the 3 tumor cells studied as well as in an iciHHV-6 individual. Interestingly, we observed integration of HHV-6A DR in the intronic region of chromosome 5q13.3 and 20q13.33, which may disturb coding potential of human genes like AGGF1 and GAIP respectively. Further studies will be required to understand these possibilities in detail. Non-telomeric integration of ciHHV-6A could be a cause or consequence of chromosomal rearrangements. Presence of homologous DR sequences at both end of the viral genome could facilitate homologous recombination between both the viral ends and thereby result in the excision of the viral genome. Under such circumstances, the broken chromosomal ends would end up in rearrangements and/or loss. Even though non-telomeric integration of ciHHV-6A appears rare, the possibility that it might play a role in disease development should be taken into consideration.

The current *in vitro* study utilises BACmid-derived HHV-6A viral particles, which carry sequence alterations that might influence viral integration. Hence, future studies should be focused on the use of non-BACmid derived viral particles that can closely resemble natural HHV-6A infections. We show experimental evidence for possible role of the host cell environment in the integration of ciHHV-6A. In order to better understand HHV-6A/B cell tropism, it may be important to study cells most susceptible to viral integration and the reason behind it.

## Material and Methods

### Ethics statement

All the iciHHV-6 and HHV-6 negative blood samples used in this study were provided by HHV-6 Foundation, USA collected with written informed consent and approved by the Ethikkommission of the University of Würzburg, Germany. All the experiments and methods were performed in accordance with the relevant guidelines and regulations of the University.

### Cell culture

HeLa (Human cervical epithelial tumor cells)^26^, SK-OV-3 (Human ovarian epithelial tumor cell, Sigma-Aldrich) and U-251 (human gliobalstoma cells, Sigma-Aldrich) were maintained in RPMI 1640, DMEM-F12 and DMEM media respectively in the presence of 10% fetal bovine serum (FBS) at 37 °C and 5% CO_2_.

### PBMC isolation

Fresh PBMCs were first separated from whole blood using Histopaq1077 solution (Sigma-Aldrich, Germany) using previously described methods^27^.

### DNA Extraction and quantitative real time PCR (qPCR)

Total cellular DNA was extracted using QIAamp DNA Mini Kit (Qiagen, Germany) following manufacturer’s protocol. For quantitative PCR (qPCR), PerfeCTa qPCR SuperMix (Quanta Biosciences) was used and PCR amplifications were done on a StepOnePlus real time PCR platform (Applied Biosciences) using manufacturer’s protocol and SYBR Green chemistry. Amplified data were analysed using StepOne Software v2.1. HHV-6 genome equivalents per cells was calculated by determining host cell number by amplification of the PI15 gene as described before^26^.

### Preparation of plasmids for standard curve analysis

Standard curve analysis for HHV-6 U94 ORF (detects both HHV-6A and HHV-6B) and human PI15 ORF were described before^26^. For standard curve analysis for HHV-6A DR and HHV-6B DR, viral DR were amplified using following primers. HHV-6ADR for 5′-CCGGCGATTCCCGGAGATGC-3′ (4279–4298, GenBank Accession X83413.1), HHV-6A DR rev 5′-CCGCGTGATTGAAGGGTGA-3′ (4416–4398, GenBank Accession X83413.1), HHV-6B DR for 5′-AAACCCTACCATCCTTCGGC-3′ (966–985, GenBank Accession AF157706.1), HHV-6B DR rev 5′-GGGACGATCCCGTTAACCAA-3′ (1113–1094, GenBank Accession AF157706.1). Amplified PCR products were agarose gel purified and were cloned into TOPO pCR 2.1 vector (Life Technologies, Germany). Cloned DNA vectors were transformed into *E. coli* DH5α and propagated to isolate ample amount of vectors for qPCR analysis. Cloned ORFs were verified by DNA sequencing.

### Pulsed field gel electrophoresis and Southern hybridisation

For analysis of chromosomal integration of HHV-6, cells were embedded into agarose moulds using CHEF Genomic DNA Plug Kits (Bio-Rad Corp., Germany). Embedded material was digested overnight with proteinase K for removal of histones and other cellular proteins. Subsequently genomic DNAs were separated by clamped-homogeneous electrical field (CHEF) electrophoresis (DRII apparatus, Bio-Rad Corp., Germany) in a 1.0% agarose gel at 6 V/cm for 24 hr with a switch time that ramped from 1 to 6 sec. 8–48 kb CHEF DNA size standard (Cat. No. 170–3707) and yeast chromosomal marker (Cat. No. 170–3605) were purchased from Bio-Rad, Germany. Southern hybridisation was performed as previously described^6, 26^. (TAACCC)_6_ oligo was used as telomere probe (Probe P4). HHV-6 viral DR specific probe was generated from a DR-PCR product (~3, 5 kb) amplified using 5′-AGGAACAGACAGACGGCCAC-3′ and 5′-CTCTGCGGGGATTCACGGAT-3′ primers. Similarly, the non-DR probe was generated using the following primers: 5′ATGAAATTATCCGGACTATACTGTG3′ and 5′GTTGGGAGTTAACATTTTGAAAGTG3′ that amplifies the HHV-6A IE2 ORF.

### Inverse PCR

Inverse PCR was adopted from previously described methods^6^ with specific modifications. Briefly, 5 μg of total cellular DNA was digested with 10 U of Mbo I (Fermentas) for 16 h. Digested DNA was purified using phenol:chloroform DNA extraction method. Half of the digested DNA was diluted with water and self-circularised by addition of 0.025 units/μL T4 DNA ligase (NEB) for 16 h at 14 °C. Ligated DNA was once again purified using phenol:chloroform DNA extraction method. 50 ng of unligated or ligated DNA was amplified for 25 cycles with Phusion high fidelity master mix with GC buffer (NEB), using inverse primer pairs that allow amplification of only re-circularised viral genome. Location of MboI cut sites of interest and possible iPCR amplification products are described in detail in Fig. 3. An additional nested PCR step was incorporated to decrease non-specific amplifications. For this, one tenth of the first PCR product was used for subsequent nested PCR for 30 cycles. Primer details for iPCR are provided in supplementary Table S3. Amplified PCR products were purified using phenol:chloroform DNA extraction method and were run on a 1% agarose gel and processed for Southern hybridisation as described above. After cloning into the TOPO pCR 2.1 vector, amplicons were sequenced by the Sanger method, using M13 forward and reverse primers (Seqlab GmbH, Germany). In several cases, purified PCR products were directly sequenced without subsequent cloning.

### Generation of clonal population of cells using Flow cytometry

Different cells were infected with BAC derived HHV-6A (U1102) at an MOI of 5. 24 hrs post infection cells were sorted on an FACS ARIA III cell sorter (BD Biosciences, Germany) and GFP positive (virus infected) single cells were collected onto 96-well plates. Clonal population of cells were grown for 10 passages after which total cellular DNA was extracted for qPCR analysis. Some of these single cell derived clones were further grown for 50–100 passages to study viral genome stability.

### Fluorescence *in situ* hybridisation (FISH) for detection of integrated viral genome

FISH analysis of viral DNA was carried out using previously described protocol^6^. HHV-6 non-DR specific FISH probes were generated by mixing purified PCR products from several different ORFs of the viral genome using primers described in this manuscript^8^ and PromoFluor-550 Nick Translation Labelling Kit (PromoKine, Cat. No. PK-PF550-NTLK-10). HHV-6 viral DR specific probe was generated by the same kit using DR-PCR product amplified using 5′-AGGAACAGACAGACGGCCAC-3′ and 5′-CTCTGCGGGGATTCACGGAT-3′ primers.

### Statistical analysis

Statistical analysis was done by an interactive model in R package that uses two- way Analysis of Variance (ANOVA) with copy number as the response. Patient ID, Sampling year and location of viral sequence (DR or entire HHV-6) were taken as factors, wherever required. Normality of data distribution and goodness of fit was assumed by observing the Residuals and Q-Q plot. Tukey’s Honest Significance Differences (HSD) test was performed for determination of individual comparisons, which calculates adjusted p-values. * < 0.05, ** < 0.01, *** < 0.001.

## Electronic supplementary material

Supplementary Information
