# Supplementary material for: Chromosomal integration of HHV-6A during non-productive viral infection
Source: Sci Rep. 2017 Mar 30;7:512. doi: 10.1038/s41598-017-00658-y (PMC5428774; doi:10.1038/s41598-017-00658-y)
Supplement: Supplementary file 1 — Supplementary Information [file 41598_2017_658_MOESM1_ESM.pdf]

Supplementary information

Title: Chromosomal integration of HHV-6A during non-productive viral infection

Article type: Original Research article

Nitish Gulve[1], Celina Frank[1], Maximilian Klepsch[1], Bhupesh K. Prusty[1#]

[1]Biocenter, Chair of Microbiology, University of Würzburg, 97074 Würzburg, Germany.

Supplementary figure legends

Fig. S1. Standard curve analysis for (a) HHV-6A and (b) HHV-6B DR specific primer pairs. Ten fold serial dilutions of HHV-6A DR (a) and HHV-6B DR (b) plasmids were tested in triplicates by qPCR. Mean Ct values were plotted against the copy numbers. Correlation coefficients and slope values are mentioned in each figure.

Fig. S2. Viral copy number as well as DR copy number analysis in 4 different cell types. (a) Different copy numbers of viral genome and DR in 35 different clones of HeLa cells are presented. (b) & (c) shows similar data from 50 different clones of SK-OV-3 (b) and U-251 (c) cells. Data represents mean copy number from 3 biological triplicates.

Fig. S3. Chromosome associated viral genome was analyzed by PFGE and FISH. (a) Presence of chromosome associated full-length viral genome as well as viral DR was studied in several HeLa clones by PFGE. C.A. chromosome associated viral genome; E.C. DNA, extra-chromosomal viral DNA. (b) Similar analysis was done using some of the SK-OV-3 clones. All the PFGE blots were first hybridized with a probe that does not detect viral DR (non-DR probe). Blots were stripped

and then probed with a DR specific probe (DR probe). Clonal populations of cells having only DR are indicated in red. HHV-6 BAC DNA is used as a positive control. (c) FISH images showing more than one chromosome associated DR in various cell types. Left panel shows FISH image from an iciHHV-6A individual whereas right panel shows the same from HeLa clone 2. DR specific staining can be seen in both telomeric as well as non-telomeric regions.

Fig. S4. DR number changes in some of the clonal population of cells during continuous cell growth whereas the viral genome remains constant. (a) One of the SK-OV-3 clone (C33) was grown for 20 passages during which total cellular DNA was collected at 9 different time points. Viral copy number and DR copy number per cell was quantified using qPCR. (b-c) Similar experiments were carried out in 2 of the U-251 clones.

Fig. S5. Inverse PCR followed by Southern hybridization detects various forms of HHV-6A genome in U-251, SK-OV-3 and HeLa cells. (a) Analysis of viral integration in U-251 and SK-OV-3 cells. First panel shows ethidium bromide (EtBr) stained agarose gel containing iPCR products. After transfer of DNA onto nylon membrane, membrane was probed with 4 different 5 prime end-labeled oligos (P1-P4). Bands detected only with probe P2 in U-251 clone 15 and 52 were sequenced for further analysis of the junction sites. (b) iPCR products from four different HeLa clones (C1-C4) were checked by Southern hybridization using two different probes (P2 and P4). ~1.5 kb amplimer is expected from a full-length viral genome irrespective of integration status. The single band detected only with probe P2 in clone 4 was sequenced for further analysis of the junction site. Potential bands that did not hybridize with a telomere probe

(Probe P4) are marked with red arrowhead and were processed for sequence identification.

Fig. S6. Sequence alignment of non-telomeric integration sites of HHV-6A as identified by inverse PCR. (a) Non-telomeric integration site at Chr 20q13.3 in U-251 clone 15. Inverse PCR derived sequence was aligned against human G-alpha interacting protein isoform B (GAIP) (GenBank accession AH010108.2) (b) Non-telomeric integration site of HHV-6A at Chr 5q13.3 in iciHHV-6A patient NNDM3. Inverse PCR derived sequence was aligned against human angiogenic factor with G Patch and FHA Domains 1 (AGGF1) (GenBank accession NG_027822.1)

Fig. S1
Gulve et al.

a

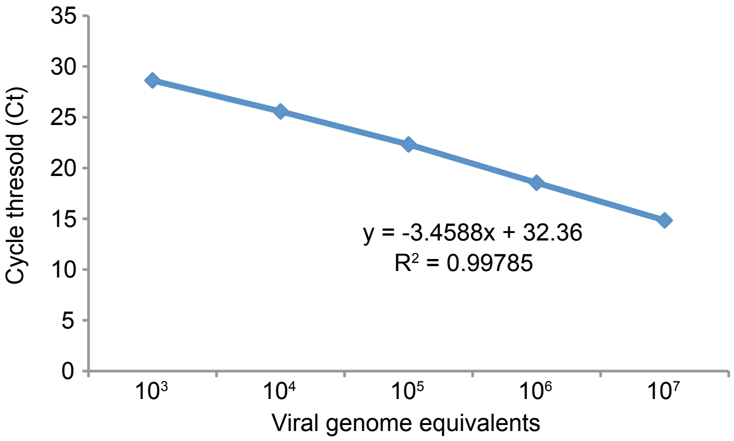

b

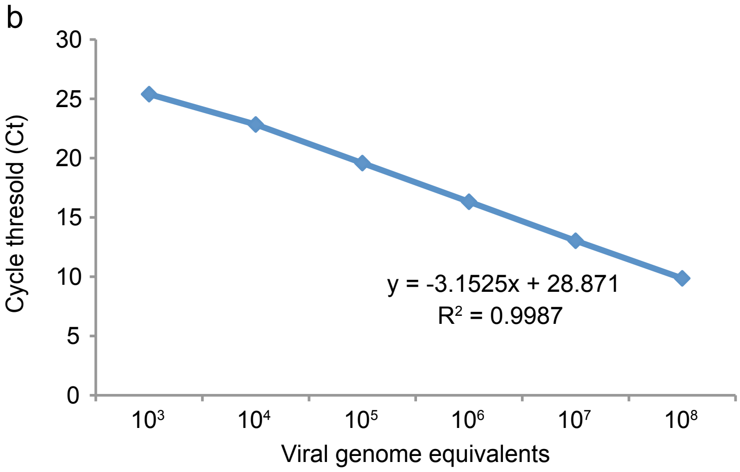

Fig. S2
Gulve et al.

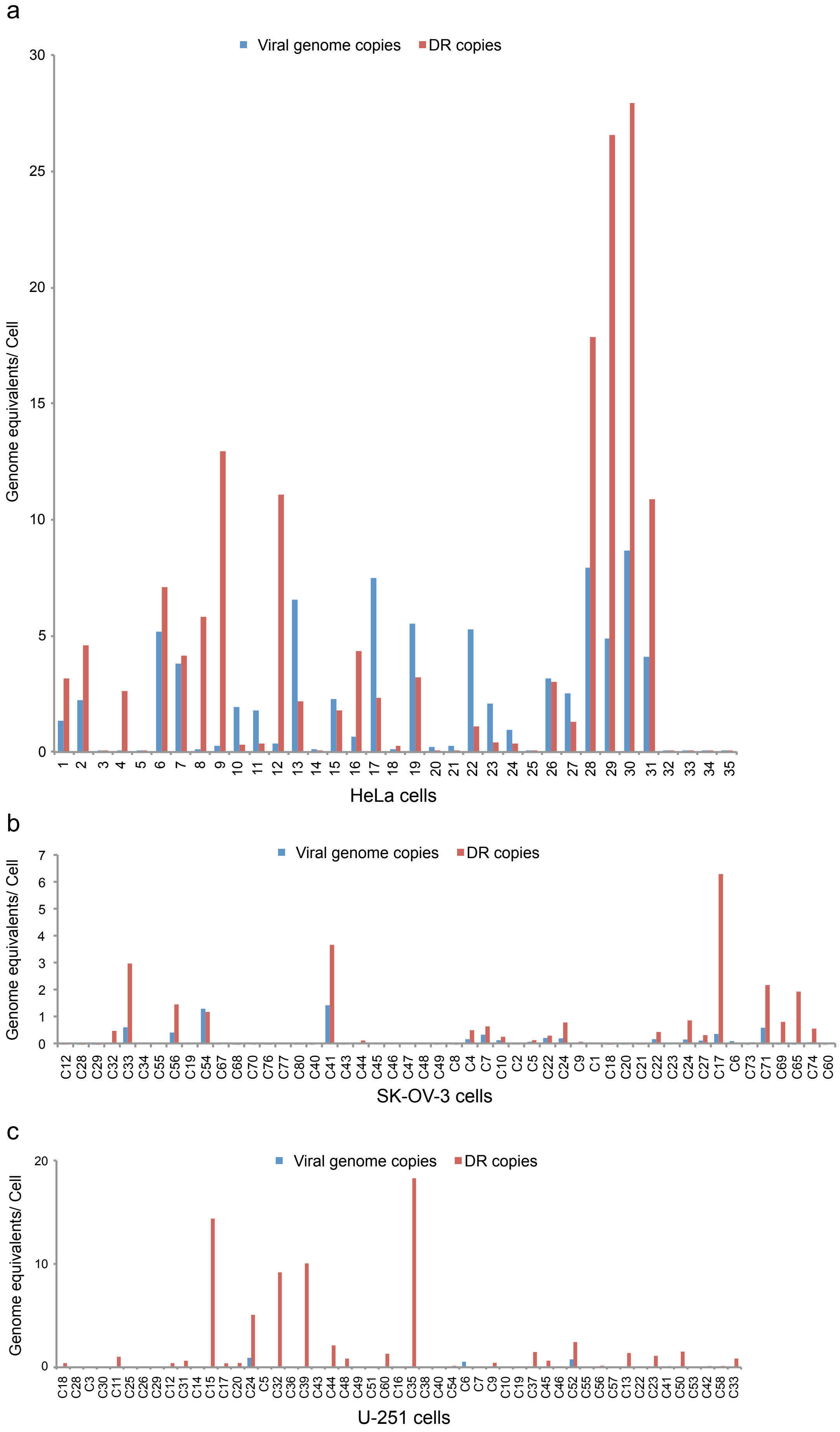

Fig. S3
Gulve et al.

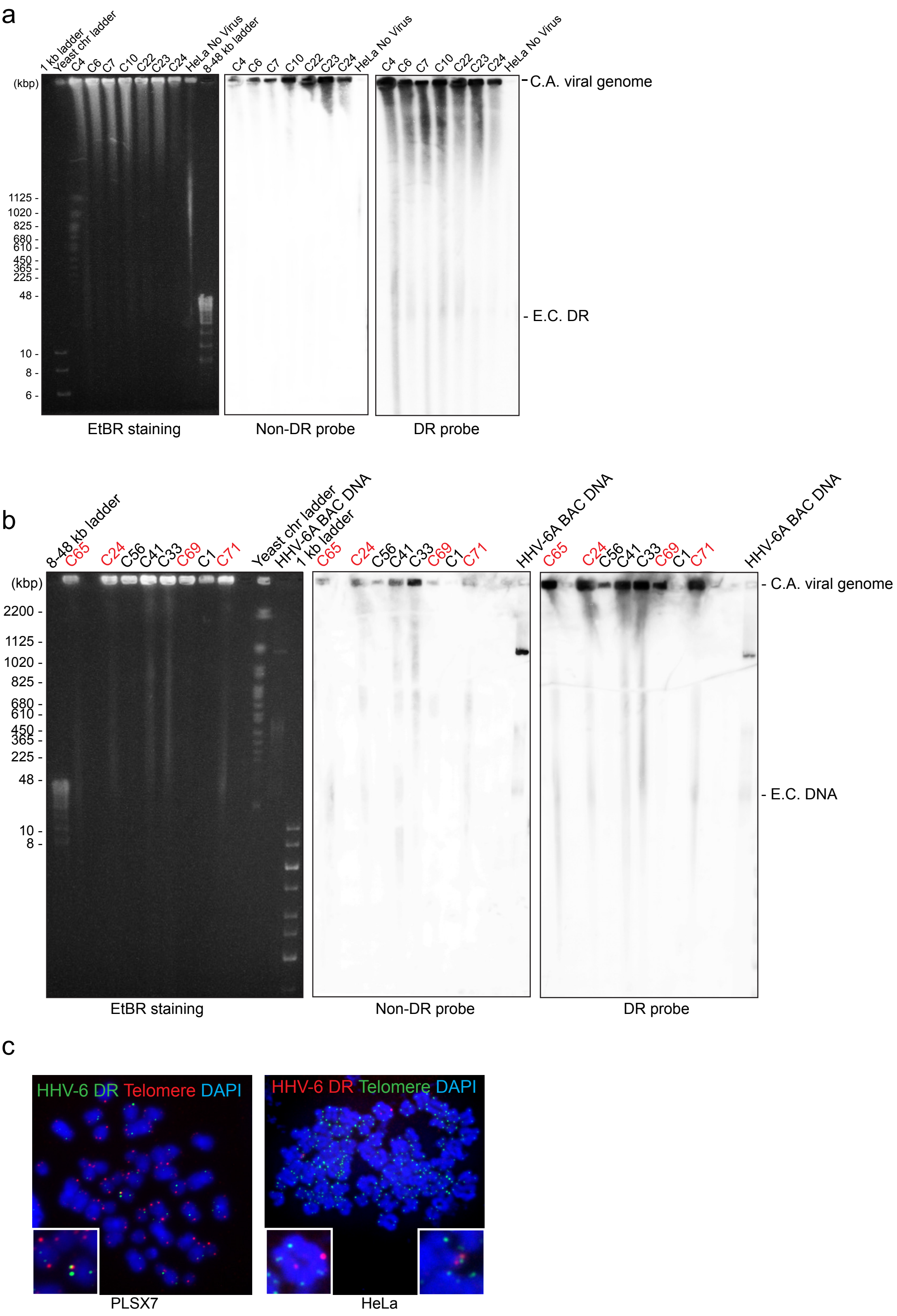

Fig. S4
Gulve et al.

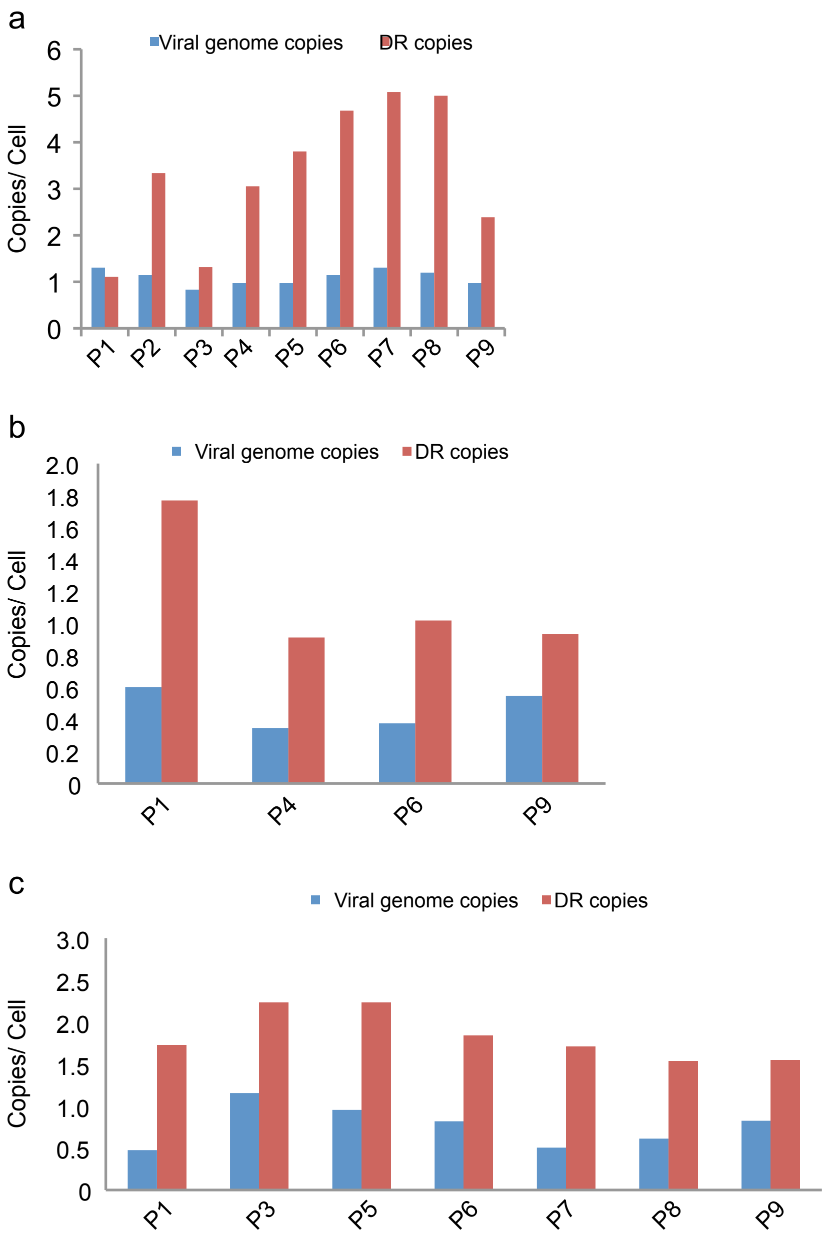

Fig. S5
Gulve et al.

a

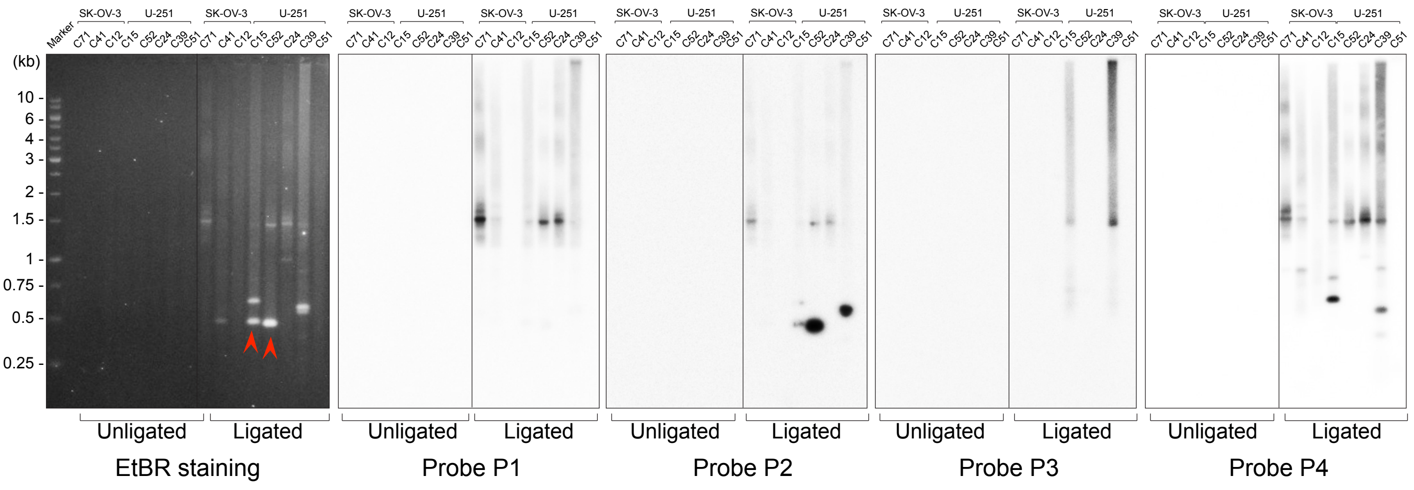

b

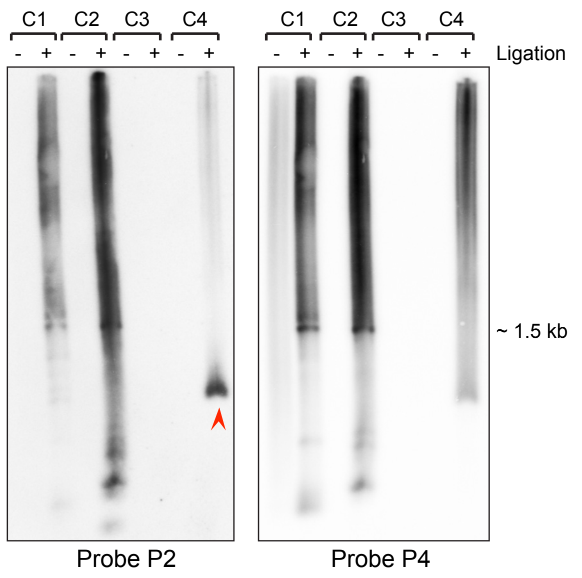

Fig. S6
Gulve et al.

a

```
U-251          -------------------------------TCTGGGACCCACAGACAGACCTGGACTCT
AH010108.2     CAGGTCTAGCCTGCTGCCCCTGGCTGAGACCTCTGGGACCCACAGACAGACCTGGACTCT
                                              *****************************

U-251          TGGTGGGAAGCCACTCTGCCCACTCCCCACCCCAGACACCCTAGAAGCCAGTCTGGCCAC
AH010108.2     TGGTGGGAAGCCACTCTGCCCACTCCCCACCCCAGACACCCTAGAAGCCAGTCTGGCCAC
               ************************************************************

U-251          CACCCGCACGCACCAGTTGGTAGCCCCAGACTCTCGGGGCCCCTTTGGGAGGGGCTGGGA
AH010108.2     CACCCGCACGCACCAGTTGGTAGCCCCAGACTCTCGGGGCCCCTTTGGGAGGGGCTGGGA
               ************************************************************

U-251          GCCGGAAACCAAGAAAGAGGAACCCAGGCTTCCGGCTTCCCAGGGAGTGAGCGGATGGGG
AH010108.2     GCCGGAAACCAAGAAAGAGGAACCCAGGCTTCCGGCTTCCCAGGGAGTGAGCGGATGGGG
               ************************************************************

U-251          GTGTCTCGGAACCTCCAGTGGGGGCCTCATCTGGGGCCCAGCCTGGGAGGAGGGGCCAGA
AH010108.2     GTGTCTCGGAACCTCCAGTGGGGGCCTCATCTGGGGCCCAGCCTGGGAGGAGGGGCCAGA
               ************************************************************

U-251          AGCTGCTGCAGCTCCCTCCTCAATGATC--------------------------------
AH010108.2     AGCTGCTGCAGCTCCCTCCTCAATGATCCTGGACCCCCTCCCAGCCCTGCTGTAAGCTTG
               ****************************
```

b

```
NNDM3          ----------------------------------------GCAGCTGCCCACTCTGAGAA
NG_027822.1    GCAACCACCCCGTCTGAGAAGTGAGGAGCCCCTCCGCCCGGCAGCTGCCCACTCTGAGAA
                                                       ********************

NNDM3          GTGAGGAGCCTCTCCGCCCGGCAGCCACCCCATCTGGGAAGTGAGGAGCGTCTCCGCCCG
NG_027822.1    GTGAGGAGCCTCTCCGCCCGGCAGCCACCCCATCTGGGAAGTGAGGAGCGTCTCCGCCCG
               ************************************************************

NNDM3          GCAGCCACCCCGTCCGGGAGGGAGGTGGGGG-----------------------------
NG_027822.1    GCAGCCACCCCGTCCGGGAGGGAGGTGGGGGGGATCAGCCCCCCGCCCGGCCAGCCGCCC
               *******************************
```

Table S1. Ct values of U94, HHV-6A DR, HHV-6B DR and PI15 qPCRs are shown in a tabular form together with the subsequent calculations and copy number analysis. Specificity of HHV-6A and HHV-6B DR primers were tested against each other and negative control samples.

Sample name	HHV-6 U94 Ct	Log10 copies	Actual viral copies	HHV-6A DR Ct	Log10 copies	Actual copies	DR-6B Ct	Log10 copies	Actual copies	PI15 Ct	Log10 copies	Actual copies	Cell number	Viral genome equivalents per cell	HHV-6A DR genome equivalents per cell	HHV-6B DR genome equivalents per cell
PLSX7	22,7648	3,457591687	2868,082816	21,761	3,064357581	11597,3184	30,056	-0,375892149	21,04155614	21,761	1,993343719	4923,95101	2461,975505	1,164951808	4,710574242	0,008546615
DSTX1	22,7986	3,447200959	2800,276779	26,7607	1,618856251	415,772969	20,5291	2,646122125	4427,128474	21,6392	2,036467922	5437,984016	2718,992008	1,029895186	0,152914377	1,628224159
NNDM3	22,7786	3,453349319	2840,20259	22,6857	2,797010524	6266,29049	30,986	-0,670896114	10,66777606	21,2247	2,183224756	7624,208422	3812,104211	0,745048517	1,643787825	0,002798396
CSSJ2	22,829	3,437855452	2740,661835	27,788	1,321845727	209,8194416	20,0157	2,808977002	6441,351551	21,675	2,023792664	5281,565477	2640,782739	1,037821777	0,079453504	2,439182692
RRCV8	22,628	3,499646469	3159,704504	22,219	2,931941714	8549,519632	29,975	-0,350198255	4,464797277	21,375	2,130009914	6744,968378	3372,484189	0,936907136	2,535080716	0,00132389
89703	20,9455	4,016877248	10396,26277	20,0984	3,545044524	35078,78351	28,7735	0,030927835	10,73810967	19,9486	2,63503753	21577,81843	10788,90922	0,963606474	3,251374425	0,000995292
93924	20,7469	4,077930462	11965,48928	20,7181	3,365878339	23220,8621	30,5844	-0,543505155	2,860848405	19,7709	2,697953548	24941,55647	12470,77823	0,959482163	1,862021893	0,000229404
63090	19,8439	4,355528913	22674,04022	28,2107	1,199635712	158,3564334	17,6469	3,56038065	36339,64248	19,9496	2,634683473	21560,23434	10780,11717	2,103320387	0,014689676	3,370987709
KHW24765	20,817	4,056380461	11386,24333	29,325	0,877471956	75,41746937	19,354	3,01887391	10444,16946	20,227	2,536467922	17196,41537	8598,207685	1,324257769	0,008771301	1,214691461
MSC9	21,4861	3,850687079	7090,666824	27,4639	1,415548745	260,3447026	18,1203	3,410214116	25716,63351	20,948	2,281192466	9553,499165	4776,749583	1,484412507	0,054502481	5,383709794
RG060716	20,7923	4,063973685	11587,07145	19,2514	3,789927142	61649,157	35,8	-2,197938144	0,063396	20,02	2,609757825	20357,6587	10178,82935	1,138350104	6,056605813	6,22822E-06
BSC11	26,7309	2,238341172	173,11758	28,0634	1,242222736	174,6717761	32,111	-1,027755749	0,938089447	20,9588	2,277368645	9469,752947	4734,876474	0,036562217	0,036890461	0,000198123
ASC25	26,8868	2,190414707	155,0296287	26,8882	1,581993755	381,9387787	34,0962	-1,657478192	0,220050221	20,605	2,402634188	12635,84216	6317,921078	0,024538076	0,060453237	3,48295E-05
922RU	31,3499	0,818377448	6,5822966	33,8199	-0,422082803	3,783704371	34,9464	-1,927168914	0,118258152	20,4668	2,451564934	14142,78498	7071,392488	0,000930835	0,000535072	1,67235E-05
935RU	31,3992	0,803221741	6,356553998	33,4104	-0,303689141	4,969478995	34,1101	-1,661887391	0,217827451	21,4519	2,102782892	6335,091563	3167,545782	0,002006776	0,001568874	6,87685E-05
DSDG	25,512	2,613052968	410,254136	28,081	1,237134266	172,6371533	35,7623	-2,185979381	0,065165933	21,4809	2,092515224	6187,072838	3093,536419	0,132616553	0,055805761	2,10652E-05

Table S2. Summary of inverse PCR results showing different combinations of HHV-6A genome detected in this study using Southern blot hybridization and a combination of 4 different probes. P1-P4, 4 different probe types used for Southern hybridization; N, negative control (HHV-6 negative).

	**Positive for Probes**	**Possible HHV-6A genome combinations**
In vitro cell line derived clones		
SK-OV-3 C70	N	Absence of HHV-6A
SK-OV-3 C33	P2	1. Non-telomeric integration of HHV-6A at DR-T2<br>2. Possibly only DR without having rest of the viral genome
SK-OV-3 C71	P1, P2, P4	1. HHV-6A genome with intact DRL-T2<br>2. DRR-T2 absent or present as a overhang
SK-OV-3 C41	P1, P2, P4	1. HHV-6A genome with intact DRL-T2<br>2. DRR-T2 absent or present as a overhang
SK-OV-3 C12	N	Absence of HHV-6A
HeLa C1	P2, P4	1. HHV-6A genome with intact DRL-T2
HeLa C2	P1, P2, P4	1. HHV-6A genome with intact DRL-T2<br>2. Non-telomeric integration of HHV-6A at DR-T2
HeLa C3	N	Absence of HHV-6A
HeLa C4	P2	1. Non-telomeric integration of HHV-6A at DR-T2<br>2. Possibly only DR without having rest of the viral genome
U-251 C15	P1, P2, P3, P4	1. HHV-6A genome with intact DRL-T2<br>2. Non-telomeric integration at DRR-T2<br>3. Presence of concatemeric or circular viral DNA
U-251 C52	P1, P2, P4	1. HHV-6A genome with intact DRL-T2<br>2. Non-telomeric integration at DRR-T2
U-251 C24	P1, P2, P4	1. HHV-6A genome with intact DRL-T2<br>2. DRR-T2 absent or present as a overhang
U-251 C39	P2, P3, P4	1. Telomeric integration at DRR-T2<br>2. Presence of concatemeric or circular viral DNA<br>3. Possible absence of DRL-T2
U-251 C51	N	Absence of HHV-6A
In vivo patient derived DNA		
CSSJ2	N	Absence of HHV-6A
NNDM3	P3, P4	1. HHV-6A genome with intact DRR-T1<br>2. Non-telomeric integration of HHV-6A at DR-T1
89703	P3, P4	1. HHV-6A genome with intact DRR-T1<br>2. DRL-T1 absent or present as a overhang
RRCV8	P3, P4	1. HHV-6A genome with intact DRR-T1<br>2. DRL-T1 absent or present as a overhang
PLSX7	P3, P4	1. HHV-6A genome with intact DRR-T1<br>2. DRL-T1 absent or present as a overhang
RMD	N	Absence of HHV-6A

Table S3: Oligonucleotides used for PCR and as hybridization probes.

Primer	Sequence (5’->3’)
Primers for HHV-6 genome analysis	
U22 For	GGATCCAAAGCAAACCAGCAAGA
U22 Rev	TGGCGGATGGCTAGTGTGCC
U42 For	AGTTAGTTTCACAGGTGTCAGC
U42 Rev	ACCGAAATCTTTCTTTTACTTGTC
U79 For	AATGGGTTCTCTAACGGTGGAT
U79 Rev	ATTCATCATGTTGTTGATCTTCGTG
U91 For	CGTTAAAGATACTGGCATGTCT
U91 Rev	TAAAGTCTCTACTGAAGAAGCA
U94 For	ACGGGGACGTGCTAATCCAT
U94 Rev	TCCGGGTGGACCGATAAAAC
DR6 For	CCGGCGATTCCCGGAGATGC
DR6 Rev	CCGCGTGATTGAAGGGTGA
DR7 For	ATGTAACCAACTCCCAGCTCGAC
DR7 Rev	GTTGGTACGTTTCCCACAGTCGT
U83 For	TATGTAGTTCCCCCGATGCG
U83 Rev	TCTGTTTTCCCAGGTACGGC
P41 For	CCTGTTTTGATGCCAACGCA
P41 Rev	AAAGCACGTTGTTGACGGTG
DRL junction For	GCACAACCCACCCATGTGGTAGTCGCGG
DRL junction Rev	TTCCATCGGTTCTTCGCGCTCAC
DRR junction for	CTACTCACCTCTGAGGCACT
DRR junction Rev	GGATTTGACGTAATTTTTAAAACGC

Oligos for iPCR

DR_L iPCR oligos

Forward Primer	TTGGTTTCCCTCAGGGTTCG
Reverse Primer 1 (R1)	CCGTTAGCGGCATCCTAGAG
Reverse Primer 2 (R2)	TGCCCGGCACGCACCGTTAG

DR_R iPCR oligos

Forward Primer	GCACAACCCACCCATGTGGTAGTCGCGG
Reverse Primer	CGTGTGTACGCGTCCGTGGTAGAAACGCG
Nested Primer 1	TGGGTACGTAGATGGGGCAT
Nested primer 2	GTGACAACGGATTACGGAGGT
Probe P1	CCATCGGTTCTTCGCGCTCAC
Probe P2	CTTACACTTGCCATGCTAGC
Probe P3	CTGTTCACAACCCTAACACT
Probe P4	CCCTAACCCTAACCCTAACCCTAA

Primers used to amplify HHV-6 DR for standard curve generation

Forward	GTCTGAATTCTGGTGGCTACTCTTGGGTCG
Reverse	AGTCGAATTCCCCCGGTATGCCCCATCTAC

Primers for HHV-6A DR and HHV-6B DR analysis

HHV-6A DR For	CCGGCGATTCCCGGAGATGC
HHV-6A DR Rev	CCGCGTGATTGAAGGGTGA
HHV-6B DR For	AAACCCTACCATCCTTCGGC
HHV-6B DR Rev	GGGACGATCCCGTTAACCAA
PI15 For	GGCGGAAGCGCTACATTTCGCA
PI15 Rev	TATTCCATATTTGCTGCCGGTGGGA
